# The effects of artificial intelligence on human resource activities and the roles of the human resource triad: opportunities and challenges

**DOI:** 10.3389/fpsyg.2024.1360401

**Published:** 2024-06-03

**Authors:** Justine Dima, Marie-Hélène Gilbert, Julie Dextras-Gauthier, Laurent Giraud

**Affiliations:** ^1^School of Engineering and Management Vaud, HES-SO, Yverdon-les-Bains, Switzerland; ^2^Department of Management, Faculty of Business Administration, Laval University, Quebec, QC, Canada; ^3^IREGE, IAE Savoie Mont Blanc, Savoie Mont Blanc University, Annecy, France

**Keywords:** artificial intelligence, human resource (HR), human resource management (HRM), HR activities, scoping review, HR tech, HR technology, HR digitalization

## Abstract

**Introduction:**

This study analyzes the existing academic literature to identify the effects of artificial intelligence (AI) on human resource (HR) activities, highlighting both opportunities and associated challenges, and on the roles of employees, line managers, and HR professionals, collectively referred to as the HR triad.

**Methods:**

We employed the scoping review method to capture and synthesize relevant academic literature in the AI–human resource management (HRM) field, examining 27 years of research (43 peer-reviewed articles are included).

**Results:**

Based on the results, we propose an integrative framework that outlines the five primary effects of AI on HR activities: task automation, optimized HR data use, augmentation of human capabilities, work context redesign, and transformation of the social and relational aspects of work. We also detail the opportunities and challenges associated with each of these effects and the changes in the roles of the HR triad.

**Discussion:**

This research contributes to the ongoing debate on AI-augmented HRM by discussing the theoretical contributions and managerial implications of our findings, along with avenues for future research. By considering the most recent studies on the topic, this scoping review sheds light on the effects of AI on the roles of the HR triad, enabling these key stakeholders to better prepare for this technological change. The findings can inform future academic research, organizations using or considering the application of AI in HRM, and policymakers. This is particularly timely, given the growing adoption of AI in HRM activities.

## 1 Introduction

The emerging ecosystem of work is changing and becoming more complex, varied, and adaptive than the traditional work environment. The workplace is transformed by emerging technologies that give rise to new ways of working. One of the most disruptive technologies of the 21st century is artificial intelligence (AI) (Wamba et al., 2021). AI is defined as “a system that can reason and learn to imitate human intelligence, particularly in repetitive, rule-based tasks, with greater precision, speed and cost savings” (Kondapaka et al., 2023, p. 283). Duan et al. (2019, p. 63) would add that “there is no commonly accepted definition of AI. It is normally referred to as the ability of a machine to learn from experience, adjust to new inputs and perform human-like tasks.” Indeed, there is a diversity of AI techniques such as, for example, machine learning (ML), deep learning (DL), or more recently popularized, generative AI (Zhuhadar and Lytras, 2023). First, ML encompasses all approaches that enable machines to learn from data without being programmed by humans to do so (Jakhar and Kaur, 2020). Second, DL is a more complex form which “involves deducing high-level abstract information from a vast dataset through machine learning” (Delbecq and Devillard, 2019, p. 13). More specifically, the algorithms mimic the architecture of the biological neural networks of the human brain (Jakhar and Kaur, 2020). Third, generative AI has gained popularity since the official release of ChatGPT in November 2022 (Budhwar et al., 2023). It is also a subfield of AI that “focuses on generating content or solutions from a model learned from data” (Marchenoir, 2023, p. 36). The aim is to enable machines to produce original and creative content, often by mimicking existing patterns and styles but going beyond prediction as done by search engines like Google (Budhwar et al., 2023). This new content created by generative AI can include text, audio, video, images, software code, and simulations (Budhwar et al., 2023). However, care must be also taken not to confuse simple automation and AI. Automation has been around for centuries and means that machines are replicating human tasks, but AI requires that the machines also replicate human thinking. In fact, automation can incorporate AI, but not necessarily (Mateu and Pluchart, 2019).

In recent years, AI has attracted increasing attention from both practitioners and researchers (Quan and Sanderson, 2018; Haenlein et al., 2019; Davenport et al., 2020; Rampersad, 2020; Robinson et al., 2020), and has become one of the most prominent research topics in business (Zhang et al., 2020). Today, AI is used and actively studied in many fields such as Health (e.g., Khalighi et al., 2024), Emotional recognition (e.g., Gursesli et al., 2024), and Education (e.g., Rahiman and Kodikal, 2024). But what do we know about the associated changes in the HR field? Human resource management (HRM) is one of the management fields that can be significantly impacted by revolutionary techniques such as AI, identified as a megatrend in this discipline (Harney and Collings, 2021). Indeed, despite the rapid growth of interest in the AI–HRM field, the academic literature related to this topic remains incomplete. In fact, the latest studies (published after 2021) have not been considered in recent reviews (Qamar et al., 2021; Budhwar et al., 2022; Gélinas et al., 2022; Vrontis et al., 2022; Basu et al., 2023; Pereira et al., 2023), and the effects of AI on the roles of the HR triad remain unknown, leaving more questions than answers (Minbaeva, 2021; Grote et al., 2023). The specific roles and shared responsibilities of each of these key actors remain unidentified, preventing them from being prepared for this technological change. For instance, HR professionals are unable to know exactly what is being disrupted by AI or how to successfully use this technology in their activities (Minbaeva, 2021).

Therefore, we raise the following question: What do we know about the effects of AI on HR activities and the roles of the HR triad? To answer this question, a scoping review of the current AI–HRM literature proves beneficial, as it offers a unique synthesis and original insights for future research (Kunisch et al., 2018; Klein and Potosky, 2019), suggesting necessary future developments (Dwertmann and van Knippenberg, 2020). Additionally, a scoping review is timely, considering that AI has reached development levels that potentially make it a valuable partner for HR (Zhang et al., 2020). Indeed, recent advancements in supercomputing power and big data technologies seem to have enhanced AI capabilities (Duan et al., 2019).

This study aims to examine the impact of AI on HR activities (both in terms of opportunities and challenges) and on the roles of each actor of the HR Triad. To this end, we grounded our work on the HR activities identified by Jackson et al. (2018), which include: (1) workforce planning; (2) job analysis and competency modeling; (3) recruitment and selection; (4) talent retention; (5) training and development; (6) performance management; (7) compensation; and (8) workplace safety, health, and wellbeing. Jackson et al. (2018, p. 19) argued that ensuring the positive, rather than destructive, effects of such HR practices necessitate the involvement of the three key players of the HR triad, “which consists of HR professionals, line managers, and all the other employees who are affected by HR policies and practices.” Therefore, our analysis focuses on the roles of these three groups particularly impacted by AI.

In doing so, this present study makes four major contributions to the field of organizational psychology and HRM. First, a core contribution of our review is mapping the developments covered in the AI–HRM literature in an integrative way, allowing for the identification of what has been studied as well as the gaps present in the scientific literature. By synthesizing and organizing existing research, this study provides a comprehensive overview of the current state of knowledge in this domain and what remains to be done. Second, this study contributes to a better understanding of the positive (opportunities) and negative (challenges) effects of AI on HR activities, which are often treated separately in the literature and are frequently juxtaposed between optimistic and pessimistic viewpoints. By examining these effects holistically, this study provides a nuanced understanding of the complex relationship between AI and HRM, offering insights into both the potential benefits and risks associated with the implementation of AI technologies in HR activities. Finally, this study contributes to the literature by identifying the effects on the roles of each stakeholder within the HR triad (employees, managers, and HR professionals). By delineating the specific impacts of AI on each group, this study provides valuable guidance for practitioners and organizations seeking to navigate the changing landscape of HRM in the era of AI. Additionally, by highlighting the roles and responsibilities of different stakeholders, this study empowers individuals within the HR triad to proactively adapt to technological advancements and leverage AI tools effectively in their respective roles.

## 2 Review methodology

We conducted a scoping review of the AI–HRM literature to uncover the contributions of this emerging subfield, which has emerged in response to the significant need for organizations to adopt AI. A scoping review is a “review that seeks to explore and define conceptual and logistic boundaries around a particular topic with a view to informing a future predetermined systematic review or primary research” (Sutton et al., 2019, p. 211). In other words, this type of review provides an initial indication of the potential size of the literature available on a particular topic (Paré et al., 2015). The main distinction from the systematic review methodology is that “scoping reviews do not aim to produce a critically appraised and synthesized result/answer to a particular question, and rather aim to provide an overview or map of the evidence” (Munn et al., 2018, p. 3). This approach is particularly useful for identifying and analyzing knowledge gaps in bodies of literature that have not yet undergone comprehensive reviews (Munn et al., 2018), typically to inform future research or policy. We conducted the systematic scoping review in line with existing guidelines (Arksey and O’Malley, 2005; Peters et al., 2015).

The present review of the AI–HRM literature seeks to answer the following research questions: How is AI affecting, (RQ1) HR activities (in terms of opportunities and associated challenges) and (RQ2) the roles of the HR triad?

### 2.1 Search strategy, article screening, and selection

To complement recent reviews, we broadened our search to include academic studies published before January 10, 2023 (the date on which we performed the data extraction) without specifying a start date. Moreover, we limited our inclusion to articles published in peer-reviewed journals to ensure a certain level of result quality (Clark and Wright, 2007). The research was restricted to four disciplines [management, HRM/industrial relations (IR), psychology, and information systems (IS)] that are likely to provide particularly relevant insights into the AI–HRM field through their complementary perspectives and their regular engagement with the HRM literature. Indeed, our intention was not only to identify conceptual gaps but also to venture into new fields that have emerged at the intersection of these disciplines.

The inclusion and exclusion criteria were determined to adequately address the initial research questions (Paré et al., 2015). For inclusion in the review, studies had to (1) focus on AI, (2) concern HR activities, (3) be related to the fields of management, HRM/IR, psychology, or IS, (4) be published in a peer-reviewed journal, (5) be published before January 10, 2023, (6) be empirical studies, and (7) be written in English. The search string was formulated to capture all terms associated with two key search topics—AI (e.g., machine learning) and HR activities (e.g., recruitment) (see Table 1).

**Table 1:** Search string used.

Search topic	Search terms*
AI technologies	AI OR “Artificial Intelligence” OR “deep learning” OR “machine learning” OR “neural network”
HR activities	HR OR HRM OR “human capital” OR “human resource*” OR “human resource management” OR “talent management” OR recruitment OR hiring OR “skill* development” OR skill* OR career OR “performance appraisal” OR “performance management” OR reward OR compensation OR “health at work” OR “safety at work” OR “well-being” OR diversity OR “industrial relations” OR “labor relations” OR “HR balanced scorecard” OR “workforce analytic*” OR “HR analytic*” OR “job design” OR “work design” OR “HR planning” OR “workforce planning” OR “coaching”

*The asterisk indicates a wildcard character to ensure that associated terms are captured in searches (e.g., plural).

**Figure 1:**
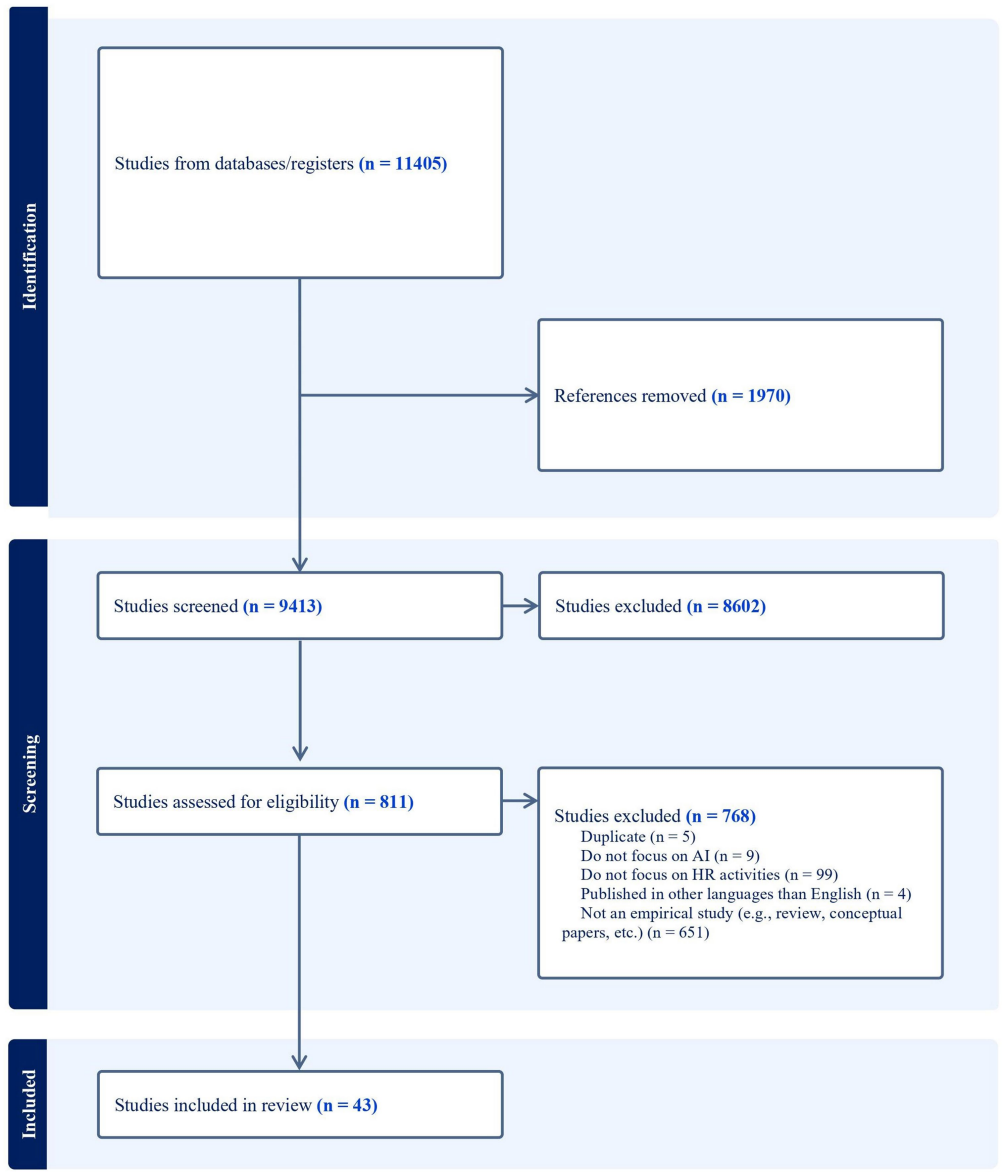
Flow diagram of the scoping review process.

To carry out the scoping review, we conducted keyword searches on titles, abstracts, and full texts in the following research databases: ABI/Inform (ProQuest), Business Source Premier (EBSCO), PsycInfo (PsycNet), and Academic Search Premier (EBSCO). We used Covidence to collect articles and to generate a flow diagram of the scoping review process (see Figure 1). Initially, 11,405 articles were retrieved from the databases for potential inclusion (1,970 duplicates were removed). A first round of selection was conducted after reviewing the titles and abstracts of 9,413 articles to remove those that did not meet the inclusion criteria. A second round of selection occurred after a full-text review of the remaining articles (*n* = 811), with 768 studies excluded for not meeting the inclusion criteria. Our final sample comprised 43 peer-reviewed articles published across 38 journals.

### 2.2 Data extraction and analysis

The first author (JD) extracted pertinent information (e.g., article references, the first author’s country of origin, research method, and sample) from the final set of 43 studies using a standardized data extraction form (Supplementary Appendix). This process provided an overview of the selected studies. The content of the selected articles was thoroughly analyzed using Max QDA. A thematic analysis of these articles resulted in their classification into eight different HR activity categories according to their topics. The second author independently coded 20% of the articles (randomly selected) to verify the quality of the first author’s coding, following recommendations by several scholars (e.g., O’Connor and Joffe, 2020). The inter-judge agreement reached 95.7%, surpassing the 85 to 90% threshold recommended by the previous literature (Miles et al., 2020). The authors discussed any remaining discrepancies to reach an agreement, allowing the first author to code the remaining articles consistently.

## 3 Results

### 3.1 Characteristics of the included studies

**Figure 2:**
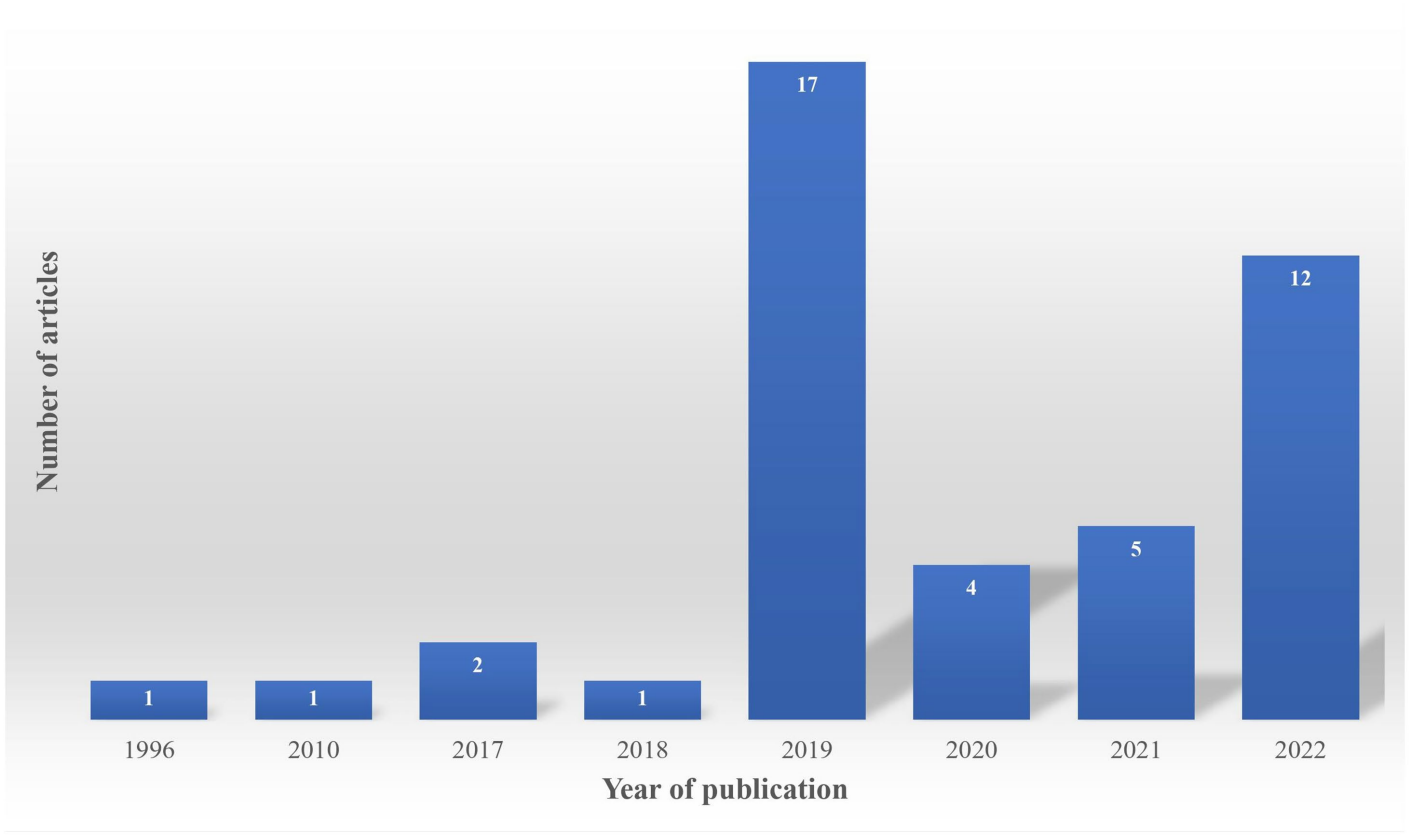
Evolution of the publications per year.

The articles included in this scoping review were published between 1996 and 2022 (Figure 2). An exponential growth in the number of articles published on this topic is apparent from 2019 onward.

**Figure 3:**
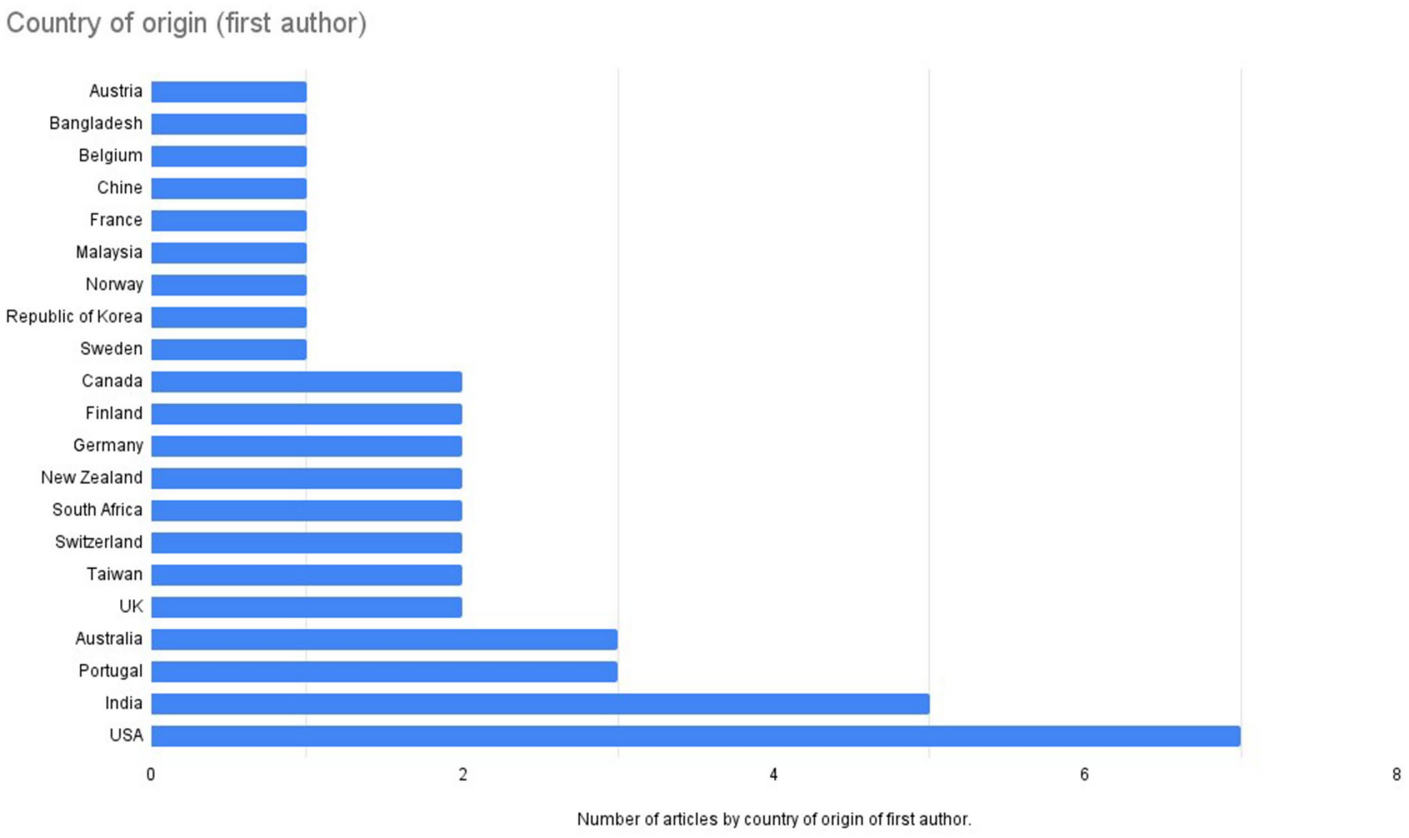
Country of origin (first author).

Among the empirical studies included, 26 are quantitative, 12 are qualitative, and five employ mixed-methods approaches (Figure 3).

**Figure 4:**
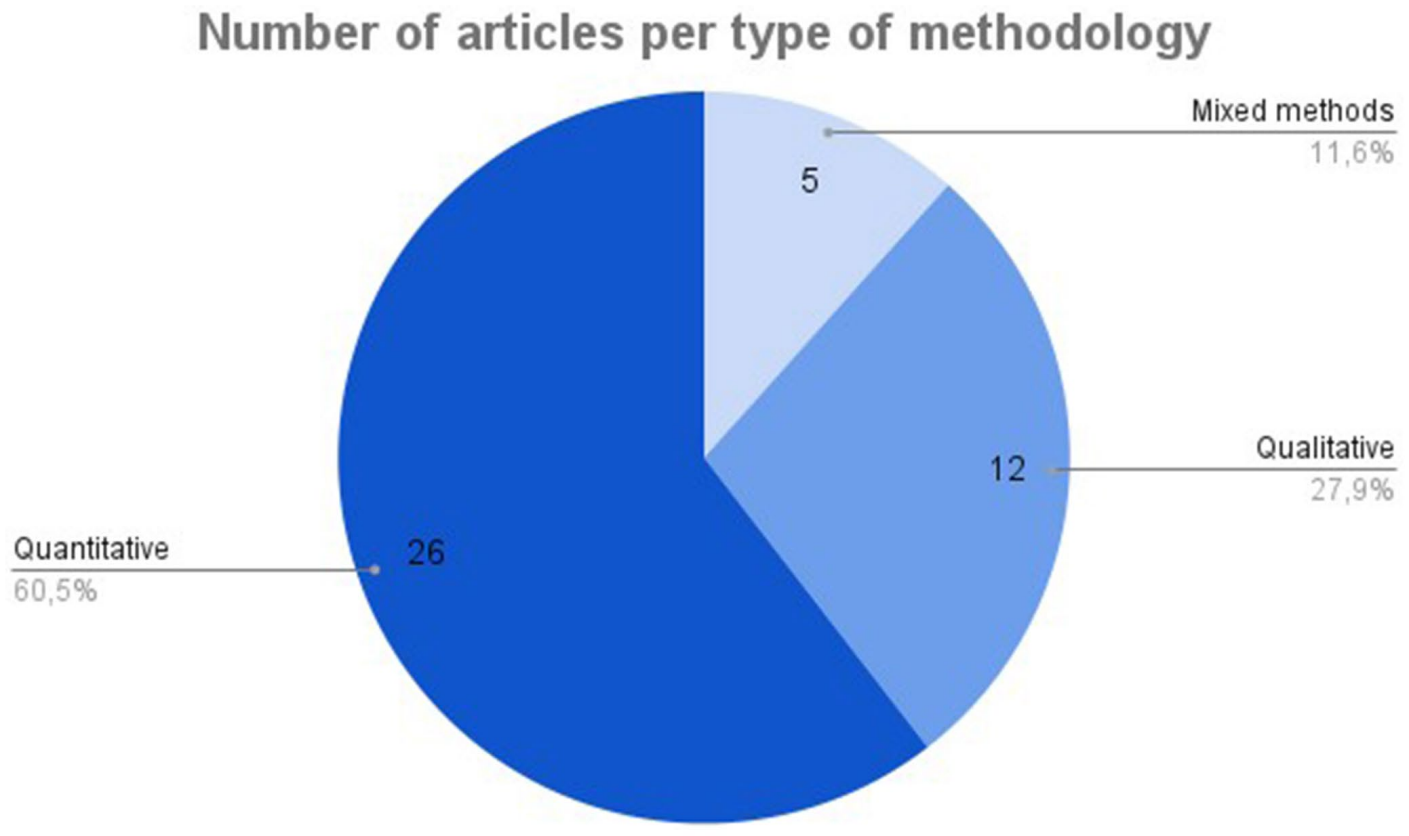
Methodology.

According to the geographic origin of the first authors (Figure 4), the majority of studies were conducted in the USA (16.3%) and India (11.6%).

**Figure 5:**
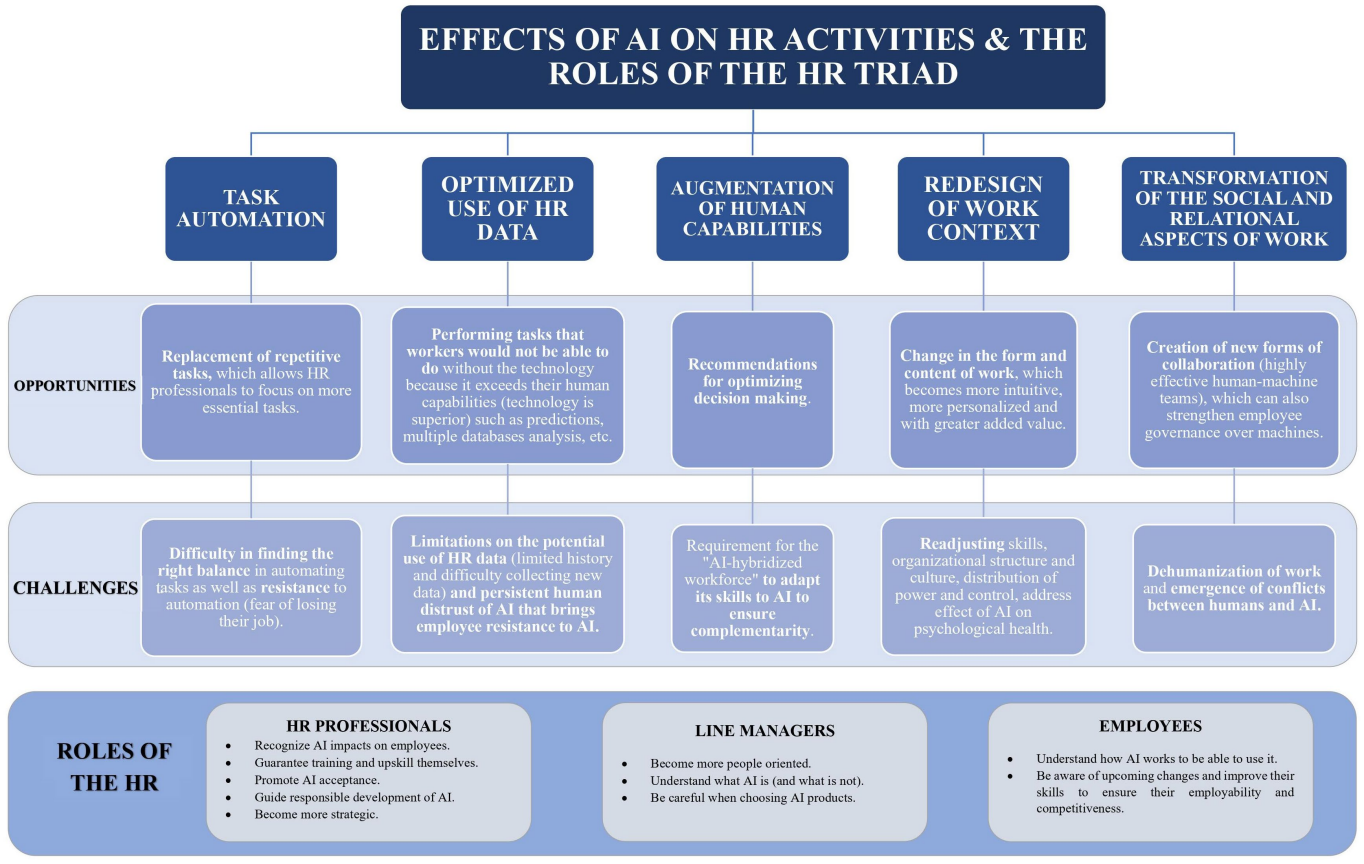
Integrative framework of the effects of AI on the HR activities and roles of the HR Triad.

The second part of our results introduces the integrative framework of AI’s effects on HR activities and the roles of the HR triad (Figure 5). This structured framework proved instrumental in attaining our research objectives and guiding future research avenues.

### 3.2 (RQ1) The effects of AI on HR activities

Our scoping review identifies five principal effects of AI on HR activities: (1) AI automates specific tasks; (2) it can optimize the use of available HR data, maximizing their utility; (3) AI likely enhances human capabilities, enabling HR specialists to perform tasks beyond their standalone capacity; (4) AI is reshaping the labor context, both in terms of work form and content; and (5) the emergence of AI transforms the social and relational aspects of work, affecting interactions and worker experience. In the following paragraphs, we will present in more detail the opportunities and challenges associated with these effects.

#### 3.2.1 Opportunities

##### 3.2.1.1 Task automation

Many opportunities associated with task automation have been identified in the reviewed literature. Among the positive impacts of AI-based technologies, the authors highlighted the replacement of repetitive tasks (Huang et al., 2019^1^; Maity, 2019; Ruckenstein and Turunen, 2020). For instance, AI can instantly filter resumes and rank the best candidates (Albert, 2019; Bongard, 2019; Chen, 2023). Benefits include reduced bias and human fatigue, improved diversity, lower costs, fewer errors, and the ability of HR professionals to concentrate on more strategic tasks (Albert, 2019; Altemeyer, 2019; Meduri and Yadav, 2021; Chen, 2023). One of the main advantages of AI in recruiting is the speed at which recruiters can respond to candidates, significantly enhancing the candidate experience (Dickson and Nusair, 2010). AI can also automate the scheduling of calls, tests, interviews, or meetings (Albert, 2019). Additionally, AI’s role in training and development can eliminate tedious tasks, such as analyzing needs assessment surveys, scheduling training programs, or manually matching trainers and trainees (Maity, 2019).

##### 3.2.1.2 Optimized use of HR data

The literature review also revealed that AI enables employees to perform tasks beyond their human capabilities, offering a technological advantage. Various authors have demonstrated that AI can predict the severity of occupational incidents (Kakhki et al., 2019), turnover intentions (Albert, 2019; Sajjadiani et al., 2019), and human performance (Sajjadiani et al., 2019), areas where HR professionals may struggle without technological assistance. AI tools can scan through multiple databases to search for candidates (e.g., LinkedIn, Glassdoor, etc.) much faster and more accurately than human recruiters (Albert, 2019). As a result, AI accelerates candidate searches, frees up recruiters’ time for more critical tasks, and improves both the quality and quantity of the talent pool (Albert, 2019; Kshetri, 2021). As a decision support tool, AI aids HR professionals in grounding their decisions on quantitative data rather than on qualitative personal judgments. During the selection process, AI can be employed in video interview analysis software to assess person–organization and person–job fit (Albert, 2019; Suen et al., 2019). AI offers opportunities to reduce bias and discrimination and to improve candidate experience (Albert, 2019; Kshetri, 2021). Indeed, AI can significantly add value to businesses by optimizing the use of HR data (Meduri and Yadav, 2021).

##### 3.2.1.3 Augmentation of human capabilities

Albert (2019) highlighted several AI applications in recruitment that enhance recruiters’ capabilities. First, the author presents a software that provides recommendations for optimizing job descriptions and tailors the language to different types of candidates (Albert, 2019). The benefits include improved diversity, reduced risk of direct discrimination, and increased candidate engagement (Albert, 2019). AI can also refine job postings by assisting recruiters in making accurate recommendations to relevant candidates (Albert, 2019).

##### 3.2.1.4 Redesign of the work context

AI is becoming an increasingly significant characteristic of today’s work environment, impacting workers (Stamate et al., 2021). In training and development, AI can transform the form and content of work, making training practices more intuitive and personalized and allowing them to be tailored to learners (Maity, 2019; Schermuly et al., 2021). Although the introduction of AI in recruitment may cause anxiety, it does not significantly affect applicants (van Esch et al., 2019). Thus, organizations need not spend money to conceal their use of AI or to reduce anxiety levels among potential candidates (van Esch et al., 2019). Instead, research suggests that organizations should promote the use of AI in their recruitment processes, which could lead to higher acceptance rates of job offers and more positive attitudes toward hiring organizations (van Esch et al., 2019). Finally, AI-augmented HRM equips employees for higher value–added jobs (Sithambaram and Tajudeen, 2022).

##### 3.2.1.5 Transformation of the social and relational aspects of work

Several reviewed articles indicate that AI is poised to create new forms of collaboration, such as highly efficient human–machine teams. Through interfaces that are yet to be designed, computers might be considered “teammates” in these novel team configurations. Notably, humans could be tasked with explaining decision-making processes, aiding machines when they encounter obstacles (e.g., due to missing data), monitoring decisions made by machines, or even training machines (Prem, 2019; Ruckenstein and Turunen, 2020). For instance, AI can also strengthen the control employees have over machines (Rombão et al., 2020), as the technology remains notably less adept at performing tasks that require human intelligence. Huang et al. (2019) call this new form of human–machine collaboration “the Feeling Economy,” which relies on typically human soft skills, such as empathy and emotional intelligence. This evolving partnership is expected to be beneficial; in fact, it could be economically advantageous for humans to manage robots. Unlike humans, robots can operate around the clock without succumbing to psychological harm (Ruckenstein and Turunen, 2020). For example, chatbots can be used in recruitment to engage candidates by providing quick responses to their queries at any time (Albert, 2019; Allal-Chérif et al., 2021). AI-based chatbots also allow employees to share their opinions and concerns, leading to better engagement (Dutta et al., 2022). Although AI can transform the relational aspect of the initial interview, AI-based interviewing is viewed as offering greater fairness, objectivity, and consistency compared to interviews conducted by humans (Kim and Heo, 2022). In training and development, AI represents an opportunity to democratize coaching in a cost-effective and scalable manner (Terblanche et al., 2022).

#### 3.2.2 Challenges

##### 3.2.2.1 Task automation

Paradoxically, while task automation offers many benefits, employees sometimes value certain repetitive tasks for the associated “brainless” time they provide, which can spur creativity (Einola and Khoreva, 2022). Consequently, finding the right balance in task automation presents a challenge. Additional challenges with task automation in recruiting include cost, privacy concerns, recruitment bias, and the potential for the replacement of recruiters (Chen, 2023). HR professionals, fearing job loss, may resist, partly due to their limited experience utilizing AI in their operations.

##### 3.2.2.2 Optimized use of HR data

A primary challenge is that HR analytics has not evolved to the same extent as it has in marketing or finance (Lismont et al., 2017), which may limit the use of HR data in AI applications. Furthermore, employees might be reluctant to be monitored or to share personal information regarding their emotions or health due to privacy concerns (Mettler and Wulf, 2019). Persistent human distrust of AI, especially in sensitive areas, such as HRM, necessitates keeping a human in the decision-making loop (Bankins et al., 2022). Paradoxically, as AI multiplies the options available, users may become overwhelmed by the perception of increased complexity (Lawler and Elliot, 1996). All of these challenges can lead to employee resistance to AI.

##### 3.2.2.3 Augmentation of human capabilities

As employees are likely to increasingly collaborate with AI, they will need to leverage its advantages while compensating for its deficiencies by developing both technical (i.e., AI knowledge and data visualization) and non-technical skills (i.e., emotional intelligence and analytical abilities) (Sousa and Wilks, 2018; Moldenhauer and Londt, 2019). In other words, the “AI-hybridized workforce” (Moldenhauer and Londt, 2019) must adapt its skills to ensure complementarity with AI.

##### 3.2.2.4 Redesign of the work context

The AI-driven redesign of the work context may necessitate skill adaptation. Consequently, intelligent machines are sometimes viewed as competitors for jobs, which fosters resistance against AI. The potential inability of workers to transfer their existing skill sets to new job requirements poses a significant challenge. Thus, retraining to acquire AI skills becomes a prerequisite for employability (Rajeshwari et al., 2019). However, it is more challenging for senior workers than for those under the age of 30 to retrain and subsequently find employment (Pulkka, 2019). Another major challenge for industries is the shortage of skilled workers (Baldegger et al., 2020) due to factors such as the retirement of baby boomers or a general disinterest in science and technology education. Moreover, our scoping review implies that AI will affect organizational cultures and structures. More specifically, AI is likely to replace certain fields of expertise, leading to changes in work and power dynamics (Sousa and Wilks, 2018), resistance to AI (Rajeshwari et al., 2019), and labor disputes (Moldenhauer and Londt, 2019). Given the rapid pace of technological change (Sousa and Wilks, 2018), developing an organizational culture that encourages continuous learning is essential for the successful implementation of AI. Additionally, AI’s impact on workers’ psychological health can be either positive or negative, depending on its perception and acceptance (Stamate et al., 2021). Therefore, addressing the challenges that AI presents is crucial.

##### 3.2.2.5 Transformation of the social and relational aspects of work

The implementation of AI-assisted HRM carries costs, including potential negative human impacts (Dickson and Nusair, 2010). Transforming the application process into a purely transactional model risks losing the nuanced analysis that a human review can provide, overcoming technology’s limitations (e.g., atypical resumes) (Dickson and Nusair, 2010). Acikgoz et al. (2020) noted that AI-based interviews are perceived as less fair, both procedurally and interactionally, compared to traditional human-conducted interviews. However, applicants have reported more issues with automated job interviews than with automated screening procedures (Wesche and Sonderegger, 2021). Chatbots may be perceived as impersonal, leading some candidates to be skeptical (Allal-Chérif et al., 2021; Kim and Heo, 2022; Weiss et al., 2022). Furthermore, AI assistance in recruitment can result in conflicts of control and power between humans and autonomous recruitment systems (Chen, 2023). To address these challenges, it seems important that policies adequately regulate the use of AI in HR (Prem, 2019).

### 3.3 (RQ2) Changes in the roles of the HR triad

The following paragraphs detail the changes induced in the roles of the HR triad.

#### 3.3.1 HR professionals

The reviewed articles suggest that HR professionals should recognize the impact of AI on employees. In this transformation of the workplace, organizations are responsible for ensuring training, internal transfers, and job placements (Sousa and Rocha, 2019; Bankins et al., 2022). Since the acceptance of AI is an essential condition for its successful implementation in organizations (van Esch and Black, 2019), HR professionals will play a decisive role in promoting this technology among employees (Islam et al., 2022). Preparing employees, managers, and themselves for the changes AI will bring is vital to preventing resistance. To this end, HR professionals should: (1) consider how to integrate AI into their business models; (2) hire technology-savvy staff to reduce resistance to change and drive innovation (Albert, 2019); and (3) allow managers and workers to familiarize themselves with potential AI-driven solutions (Kolbjørnsrud et al., 2017). HR professionals must understand the opportunities and challenges of AI-enabled HRM to leverage this technology wisely (Dickson and Nusair, 2010). They also need to update their skills, capabilities, and competencies (Nankervis et al., 2021). For example, the role of training designers becomes more strategic, as AI’s involvement could render decisions about training needs, delivery, and trainer selection more prescriptive or suggestive (Maity, 2019). Finally, HR professionals play an important role in guiding the development of AI toward responsible and less biased outcomes (Wesche and Sonderegger, 2021), sharing their knowledge with AI developers throughout the technology’s development (Soleimani et al., 2022).

#### 3.3.2 Line managers

Managers must adapt to AI, as it will significantly alter both their work and that of their direct reports (Kolbjørnsrud et al., 2017). Empirical evidence suggests that with advancements in AI, managerial roles tend to shift toward being more people-oriented, with less emphasis on thinking-focused tasks (Huang et al., 2019). As their roles evolve alongside emerging technologies, line managers also need to understand what AI can and cannot do (Rajeshwari et al., 2019). This knowledge will help them identify how AI can add value to their businesses (Rajeshwari et al., 2019). Albert (2019) offered several pieces of advice to managers on their role in integrating AI. First, managers should exercise caution when choosing AI products due to potential technical issues or compatibility problems with the organization, thus necessitating thorough evaluation before purchase (Albert, 2019). Managers should also embrace AI adoption promptly, as there are disadvantages not only for early adopters but also for late adopters, who may find it more challenging to catch up (Albert, 2019). However, managers’ readiness and enthusiasm for AI vary considerably across organizational levels and countries, which can greatly limit their organizations’ ability to adopt AI (Kolbjørnsrud et al., 2017).

#### 3.3.3 Employees

AI is increasingly integrated into HR activities, whether candidates like it or not, and is revolutionizing the way they must present themselves, which appears to be particularly beneficial for minorities and disadvantaged groups (Albert, 2019). Employees need to understand how to appeal to both machine and human interviewers (Dickson and Nusair, 2010). In essence, they are responsible for deciphering what the system is looking for to increase their chances of being hired (e.g., by adopting appropriate body language during video screenings). The role of employees involves becoming more aware of possible changes (Dickson and Nusair, 2010) in the work context in order to improve their skills in line with these developments (Jaiswal et al., 2022), thereby remaining competitive in the labor market.

## 4 Discussion

### 4.1 Theoretical implications and agenda for future research

In conjunction with previous investigations specifically addressing AI and HRM (e.g., Parry and Battista, 2019; Kaur et al., 2021; Qamar et al., 2021; Tuffaha and Perello-Marin, 2021; Votto et al., 2021; Budhwar et al., 2022; Garg et al., 2022; Gélinas et al., 2022; Palos-Sánchez et al., 2022; Vrontis et al., 2022; Pereira et al., 2023; Alsaif and Sabih Aksoy, 2023; Basu et al., 2023; Bujold et al., 2023; Chowdhury et al., 2023; Jatobá et al., 2023; Kaushal and Ghalawat, 2023; Malik et al., 2023; Pan and Froese, 2023; Prikshat et al., 2023), we provide several contributions to the academic field. First, to our knowledge, our scoping review is the most inclusive so far, as we expanded our data extraction across four disciplines (management, HRM/IR, psychology, and IS). Moreover, our review is unique in covering an extensive period (1996 to January 2023). Beyond its temporal scope, a core contribution of our review is mapping the developments covered in the AI–HRM literature in an integrative way. The proposed integrative framework identifies five main effects of AI on eight existing HR activities, as well as the associated opportunities and challenges. This represents a distinct contribution of our scoping review, since it is the only one to utilize these eight HR activities proposed by Jackson et al. (2018), offering a comprehensive overview of the field. Hence, we contribute to the literature on AI-augmented HRM, which lacks a theoretical foundation and remains fragmented and incomplete (Prikshat et al., 2021). Our data provide valuable insights into how HRM is likely to evolve, suggesting new directions for future research. We also note that the methodology of the scoping review serves as an initial step prior to conducting a systematic review (Munn et al., 2018). In this connection, the results of our work can inform the formulation of specific questions for future systematic reviews.

Our study’s results can contribute to the development of several key HRM theories. On the one hand, the research paths we propose relate specifically to theories that focus on the individual level. First, the conservation of resources (COR) theory could be used to analyze the effects of AI on HR activities. The various opportunities associated with each of the five effects of AI on HR activities presented in our results can be analyzed from a resource perspective. For instance, AI’s role in automating routine tasks raises the following question: Does AI serve as an additional resource for the key players in the HR triad? In this context, the job demands–resources (JD-R) theory could also be employed to assess both resources (opportunities identified) and demands (challenges identified). This theory could help explore the impact of AI usage in HR activities on the wellbeing and job performance of the HR triad. Furthermore, the introduction of AI is likely to influence the distribution of power by modifying the roles and responsibilities of the HR triad’s key actors. Power theory may prove helpful in understanding how these changes affect power dynamics within organizations and the implications for power relations. Do these changes introduce new demands or resources? Will business experts be sidelined in favor of data scientists? As shown by our results, the integration of AI may also lead to changes in job roles. Employing identity theory could be valuable in investigating whether the advent of AI in HR activities alters the identities of the HR triad’s key players. If so, does this change in identity constitute a demand or a resource? These changes in roles and responsibilities may also raise issues related to meaning and the identities of these actors. Sensemaking theory could offer valuable insights into how the HR triad actors interpret these changes and make sense of the impact of AI on their roles. Furthermore, the integration of AI into HR activities may require employees to acquire new skills. Human capital theory supports the notion that ongoing investments in training and development are crucial to adapting to technological advancements and maximizing the potential benefits of the technology. Therefore, exploring this research avenue using this theory could shed light on the unique implications of AI. Motivation theory posits that employees are motivated by job enrichment and opportunities for skill development (Gould, 2024). Applying this theory could reveal how AI can enhance, rather than impede, job roles, considering that without motivated and well-trained employees, organizations are likely to fail in implementing AI successfully. Furthermore, the integration of digital solutions can damage employees’ perceptions of their skills and autonomy at work (Shulzhenko, 2024). Thus, empirical research is needed on how AI impacts employees’ satisfaction and needs (Shulzhenko, 2024), which could be accomplished with the help of self-determination theory.

On the other hand, certain research paths pertain specifically to organization-centered theories. For example, future studies should employ contingency theory to examine how organizational structures can adapt to remain competitive and sustainable in response to the use of AI in HRM activities. In fact, contingency theorists argue that the most effective organizational structure depends on various factors, such as technology and the external environment (Garavan and O’Brien, 2024). At the same time, incorporating AI into HR activities may require adjustments to the organizational structure, a topic that warrants further investigation. Finally, another avenue of research could be specifically applying the adoption of AI as a source of work transformation within the emergent theoretical model of HR ecosystem alignment proposed by Yalenios and d’Armagnac (2023), who also employ contingency theory. This approach would enable researchers to better understand the dynamic effects of this technological change in the HR ecosystem.

This scoping review has highlighted current gaps in the scientific literature. For example, future research should focus more closely on the effects of AI implementation on occupational health (e.g., on workplace wellbeing and distress). Further investigation is also needed regarding the effects of AI on other HR activities, such as performance management, talent retention, and compensation. More generally, it would be interesting to explore whether AI will redefine HR activities, the processes involved, and the sectors most affected.

Empirical research is essential for gaining a deeper understanding of the five effects of AI on HR activities identified in this scoping review. For example, how can the right balance in automating tasks be achieved? What are the consequences of replacing repetitive tasks? (E.g., Is the level of fatigue higher? Is employee satisfaction higher?) Regarding the optimized use of HR data, the international HR tech market was estimated at $32.58 billion in 2021 and is expected to reach $76.5 billion by 2031, which represents a growth of 9.2% over a decade (Research and Markets, 2023) and shows significant market interest. The pressing questions now include the extent to which companies can utilize HR data, what employees are willing to accept in this context, and how the legal aspects of this usage can be delineated. Although AI has demonstrated its ability to augment human capabilities, more research is needed to better understand the complementarity between humans and machines. In fact, this complementarity is essential because, as Hamouche et al. (2023) pointed out, it could threaten the sustainability of employees’ skills and future career prospects. Another complex challenge for organizations is the redesign of the work context. There is a need for more research on how organizations can adapt their structures, cultures, and power distributions. Indeed, several studies (e.g., Di Vaio et al., 2020; Zarifis and Cheng, 2023) have shown that the changes brought about by AI can significantly impact organizations’ business models. More investigation into this area is recommended, as suggested by Budhwar et al. (2023). Furthermore, the specific effects of AI on HR business models require further exploration, although some research groups have begun to examine this issue (e.g., Minbaeva, 2021). Understanding how employees perceive personalization (whether as genuine added value or overly intrusive) is also crucial. Additionally, the transformation of the social and relational aspects of work, identified in this scoping review, deserves more attention. Questions surrounding the dehumanization of work, for instance, would benefit from further investigation. Ultimately, the inquiry into whether AI will redefine HRM—and if so, how—remains open.

Recent reviews have investigated AI in the HR context, yet none focused on the interplay between AI, HR activities, and the HR triad. The reviewed literature appears to overlook how AI affects employees’ roles, even though this group may be critical for AI’s successful implementation. Our review aggregates existing knowledge on this topic by identifying the effects on the roles of each stakeholder within the HR triad (employees, managers, and HR professionals). This integrative synthesis notably shows how AI innovations can either augment the knowledge and skills of HR triad actors or render them obsolete (Paschen et al., 2020). Furthermore, future research should examine workers’ apprehensions and expectations concerning AI to better structure AI–human collaboration and management strategies for successful AI implementation. Conducting case studies on organizations that showcase their HR strategies in relation to AI would enrich the AI–HRM interface literature by documenting effective practices and pitfalls, offering valuable lessons for other organizations. As AI becomes increasingly integrated into the workplace, supporting employees through the ensuing changes will be crucial for organizations. Many questions remain, such as how managers should address employee resistance to AI, whether this resistance differs from that encountered with previous technologies, and why. Additionally, research is needed to elucidate the factors contributing to the social acceptance of AI at work. Future studies should attempt to explain AI’s impact on leadership and managerial roles, including how managers adapt to sharing leadership and decision-making with AI.

Our scoping review indicates a shift toward a more strategic role for the HR function. The literature reviewed suggests that HR practitioners are tasked with facilitating the social acceptance of AI, ensuring its ethical use, managing new hybrid teams (human–machine), and upskilling employees (as well as themselves). HR professionals play a pivotal role in ensuring the effective application of AI in HRM. Their centrality is underscored by the need to bridge the gap between individuals, businesses, society, and governments regarding AI (Minbaeva, 2021). Many questions remain, particularly regarding how HR practitioners will align the diverse stakeholders (managers, employees, etc.) involved in AI-augmented HRM. Scholars might explore how the four roles of HR professionals outlined in Ulrich (1996) recognized HR model (strategic partner, change agent, administration expert, and employee champion) evolve with AI integration. There could be connections between these roles and the impacts of AI on HR activities identified in our scoping review, such as task automation’s link to the administration expert role. Is AI transforming the HR profession or merely evolving it? If so, how? Future research could also examine how HR professionals might steer the responsible development of AI. Is it a matter of including an HR professional in the technology development team? What are some other ways of accomplishing this? What criteria should HR professionals consider to ensure that AI is developed responsibly? Further study is needed to shed light on how the HR triad responds to these accumulated challenges, as our data suggest that HR professionals may also need to address their own resistance to AI. Therefore, organizational change management may emerge as one of several critical skills for successfully integrating AI in the workplace (Giraud et al., 2022). More research is required to comprehend how HR professionals can identify AI’s impacts on employees and support them through this technological transformation.

Given the potential for AI to be a particularly disruptive technology (Harney and Collings, 2021), further empirical investigations of its individual and collective effects appear necessary. To this end, our scoping review contributes original insights by examining the various opportunities and challenges AI presents for each actor in the HR triad. Minbaeva (2021) argued that focusing solely on individuals might be too narrow a perspective, urging HRM researchers to consider broadening their scope to include interactions between machines and individuals. Indeed, the process through which AI and HR may be able to cooperate appears to be the principal factor in successful AI implementation (Makarius et al., 2020). Acknowledging the HR ecosystem and other environmental factors “appears crucial when explaining HRM practitioners’ roles” (Vincent et al., 2020, p. 465), notably when it comes to AI implementation. For this line of research, focusing on the potential role of unions—considering their significant influence in the HR ecosystem, yet their absence in the reviewed literature—could provide valuable insights into the use of AI in HR, its impact on employees, and its ethical use. Multilevel investigations of the AI–HRM literature are therefore promising research paths, notably to elucidate the mechanisms or “black box” through which HR practices alleviate or aggravate AI impacts on individuals, teams, and organizations. Since AI is set to become a partner at work, gaining a better grasp of how this technology affects teamwork becomes imperative. As suggested by Grote et al. (2023), future research should focus on AI as teammates, human–AI team processes and emergent states, and human–AI team effectiveness. Analyzing the differences between human–AI teams compared to human-only teams would also be important. Such research would help to better introduce human–AI teaming into organizations. Although several authors (Plastino and Purdy, 2018 Britt, 2019) recommend an organizational culture open to AI, no identified study truly describes what characterizes this type of culture or how to implement it. Finally, future studies should distinguish the differing challenges AI in HR presents for large organizations versus SMEs.

The reviewed articles confirm that ethical concerns often arise with the organizational use of AI (Malik et al., 2020). Thus, our work contributes valuable insights into theoretical reflections on AI ethics in organizational contexts (Dwivedi et al., 2021). As the way to bridge legal gaps regarding the misuse of AI is still under debate (Buchholtz, 2020) and remains to be addressed by incoming regulations, companies may have to bear the responsibility for the ethical implementation of AI (Helbing, 2019), at least temporarily.

In conclusion, our reflections led us to propose a research agenda that addresses the gaps identified from the current state of knowledge (Table 2).

**Table 2:** Future research agenda for the AI-HRM field.

Theoretical development	Would AI redefine HRM and how?
Effects of AI on HR activities	Would AI redefine HR activities, how and in which sectors?
	How can the right balance be found in automating tasks? What are the consequences of replacing repetitive tasks (e.g., Is the level of fatigue higher? Is employee satisfaction higher?)? Does AI dehumanize work?
	What are the effects of AI on occupational health? How can AI improve employee mental health at work?
	What are the effects of AI on performance management?
	What are the effects of AI on talent retention?
	What are the effects of AI on compensation?
Effects of AI on the roles of the HR triad	How do the members of the HR Triad manage the work with AI-augmented HRM?
	How do line managers perceive the sharing of decision-making and power with AI?
	Would resistance to AI be different from resistance to former technologies and why?
	What are workers’ apprehensions and expectations of AI? How personalization is perceived by employees (is it a real added value or is it too intrusive)?
	How should managers and HR professionals handle employee resistance to AI?
	How is AI changing the four roles of the HR professionals (strategic partner, change agent, administration expert, employee champion)?
	How HR professionals might guide the responsible development of AI? What are some other ways of doing this? What criteria should HR professionals use to consider that AI is being developed responsibly?
	How HR practitioners are going to be able to align the multiplicity of stakeholders (managers, employees, etc.) involved in the AI-augmented HRM?
Effects of AI on teams	How can the integration of AI as teammates be optimized to enhance collaboration, understanding, and overall effectiveness within human-AI teams?
	What are the differences between human-AI teams compared to human-only teams?
	How to introduce human-AI teaming in organizations?
Effects of AI on the HR ecosystem	What is the role of organizations in helping the HR Triad to face the various challenges which were identified in the present article?
	How can organizations foster an organizational culture that facilitate the integration of AI? How organizations can adapt their structure and the distribution of power?
	What are the environmental factors to consider when studying the impact of AI on the HR ecosystem?
	What is the role of unions in the use of AI in HR?
	How far can companies go with the use of HR data? What are employees prepared to accept in this regard? How can the legal aspect of this use be framed?
	What are the differing challenges of AI in HR between large organizations and SMEs?

### 4.2 Practical implications

The present study also has considerable implications for practitioners. To foster corporate innovation, creativity, and improved AI applications, we encourage organizations to participate in upcoming research efforts in the AI–HRM field to leverage evidence-based management.

Given the ongoing development of AI, our results suggest that members of the HR triad need to regularly upskill to cope with AI integration effectively. This adaptation demands a robust hybrid HR approach (Makarius et al., 2020). Therefore, the attraction and retention of employees and the roles of managers and HR professionals who are receptive to AI adoption could be critical for organizations seeking a sustainable competitive edge.

This study provides evidence that strengthens the legitimacy of HR professionals in AI-related transformations. HR professionals can facilitate AI adoption by training employees to utilize AI-powered tools and systems and collaborating with other departments (e.g., IT) to ensure seamless integration of AI solutions within the organization. However, the dual role of HR professionals—as both employees and HR specialists who support management—may complicate their ability to lead the technological change if they are not personally convinced of its benefits. Just as some companies have begun allocating resources to organizational change management in recent decades, we recommend that concerned organizations explicitly incorporate the strategic goal of AI adoption into the missions of their HR departments or establish dedicated units. These units would bring together not only HR specialists and organizational psychologists but also IT specialists and data scientists.

AI adoption also provides HR professionals the opportunity to assume a more strategic role within organizations by leveraging data-driven arguments. For instance, they could justify the need for investment in a specific HR activity (e.g., training) using data that quantify their arguments (e.g., “50% of workers with unique skills will leave the company within 5 years”). Another strategic decision-making process that could be undertaken by HR professionals is the evaluation of different AI solutions available on the market to determine whether they meet the organization’s needs, considering factors such as cost, ease of use, data privacy, and security. However, for this to be feasible, HR professionals will need to develop technical skills that are currently far from widespread.

Additionally, our study shows that organizations need to consider the responsibilities associated with the ethical use of AI. Again, HR professionals in particular should be actively involved in this area, as they already deal with ethical and legal issues within organizations. This includes ensuring that AI systems are transparent and unbiased and comply with all laws and regulations. They should also ensure that the use of AI aligns with the organization’s values.

### 4.3 Limitations

Despite the strengths of the scoping review methodology, it is also important to acknowledge its limitations. First, like any scoping review (Grant and Booth, 2009), our work does not include a process of quality assessment. Indeed, a scoping review aims at providing a preliminary assessment of the potential size and scope of the available research literature (Grant and Booth, 2009). Second, the scope of our review is restricted to the selected keywords. Future research may choose to include more. Third, the articles included in this scoping review were sourced from four databases corresponding to four disciplines. Exploring additional databases and disciplines could reveal further contributions to the AI–HRM literature.

## 5 Conclusion

Drawing on empirical articles within the AI–HRM field, this scoping review outlines the current state of knowledge to further advance this field. Our work introduces an integrative framework detailing AI’s effects on HR activities and the roles of the HR triad. Our data reveal that the reviewed articles extensively cover five primary effects of AI on HR activities: task automation, optimized use of HR data, augmentation of human capabilities, redesign of the work context, and transformation of the social and relational aspects of work.

Our analysis provides a comprehensive overview of the evolving landscape of HR activities in the era of AI. Overall, AI is reshaping HR by offering powerful tools that enhance the efficiency, decision-making, and employee experience of HR professionals. When properly implemented and balanced with human intervention, AI can become an invaluable asset in achieving organizational objectives.

However, it is important to ensure that AI is utilized ethically and responsibly and that it complements rather than substitutes the human aspect of HR. The goal is to deploy AI within the HR profession and its served populations sustainably.

To conclude, we call for further empirical investigations into AI adoption in HRM to deepen our understanding of this topic and enable scholars to assist employees, line managers, and HR professionals in the positive and sustainable implementation of AI in organizations.

## Data availability statement

The original contributions presented in the study are included in the article/Supplementary material, further inquiries can be directed to the corresponding author.

## Author contributions

JD: Conceptualization, Data curation, Formal analysis, Funding acquisition, Investigation, Methodology, Project administration, Resources, Software, Supervision, Validation, Visualization, Writing – original draft, Writing – review & editing. M-HG: Conceptualization, Data curation, Formal analysis, Funding acquisition, Investigation, Methodology, Resources, Software, Supervision, Validation, Writing – original draft, Writing – review & editing. JD-G: Writing – original draft, Writing – review & editing. LG: Writing – original draft, Writing – review & editing.

## Footnotes

^1^Included articles are identified by an asterisk.
